# Accuracy of Length of Virtual Stents in Treatment of Intracranial Wide-Necked Aneurysms

**DOI:** 10.1007/s00270-019-02230-9

**Published:** 2019-05-10

**Authors:** Kengo Nishimura, Katharina Otani, Ashraf Mohamed, Chihebeddine Dahmani, Toshihiro Ishibashi, Ichiro Yuki, Shogo Kaku, Hiroyuki Takao, Yuichi Murayama

**Affiliations:** 1 Division of Endovascular Neurosurgery, Department of Neurosurgery, School of Medicine, Jikei University, 3-25-8 Nishi-Shimbashi, Minato-ku, Tokyo, 105-8461 Japan; 2 Siemens Healthcare K.K., Gate City Osaki West Tower, 1-11-1 Osaki, Shinagawa-ku, Tokyo, 141-8644 Japan; 3 Siemens Healthcare Pte Ltd, Surgery Business Line, 60 MacPherson Road, Singapore, 348615 Singapore

**Keywords:** Stent simulation software, Stent-assisted coiling, 3D digital subtraction angiography, Wide-necked aneurysm

## Abstract

**Background and Purpose:**

Precise stent deployment is important for successful treatment of intracranial aneurysms by stent-assisted coiling (SAC). We evaluated the accuracy of virtual stents generated using commercial stent planning software by comparing the length of virtual and actually deployed intracranial laser cut stents on three-dimensional digital subtraction angiography (3D-DSA) images.

**Methods:**

We retrospectively analyzed the data of 75 consecutive cases of intracranial wide-necked aneurysms treated with the SAC technique using laser cut stents. Based on 3D-DSA images acquired by C-arm CT, stent sizing and placement were intraoperatively simulated by a commercial software application. The difference in length of the stents was estimated by measuring proximal discrepancies between the end points of the virtual and actually deployed stents on fused pre-procedural and post-procedural 3D-DSA images. Discrepancies between distal stent end points were manually minimized. The Kruskal–Wallis test was applied to test whether stent location, type, and length had an effect on difference in length between virtual and real stent.

**Results:**

The median difference in length between virtual and real stents was 1.58 mm with interquartile range 1.12–2.12 mm. There was no evidence for an effect of stent location (*p *= 0.23), stent type (*p *= 0.33), or stent length (*p* = 0.53) on difference in length between virtual and real stents.

**Conclusions:**

Stent planning software allows 3D simulation of laser cut stents overlain on 3D-DSA images of vessels and may thus be useful for stent selection and deployment of laser cut stents during stent-assisted coiling of intracranial aneurysms.

## Introduction

Although braided stents including flow diverters are increasingly used for the treatment of wide-necked aneurysms of a diameter > 10 mm [1, 2], stent-assisted coiling (SAC) remains a common treatment option for most cases [3–7]. SAC combines aneurysm coiling with parent artery reconstruction with a stent [8, 9]. It is commonly used in the treatment of simple sidewall saccular aneurysms, bifurcation aneurysms, fusiform aneurysms, and aneurysms with a poor dome-to-neck ratio, a wide neck, or complex anatomy [10].

The success of SAC depends on selecting the optimal length and diameter of the stent and on its correct positioning [11, 12]. If the stent is too long, the risk of infarction increases, because the stent may potentially block perforator arteries [9, 13]. Longer stents are more difficult to fit because they may traverse multiple curvatures. From a procedural point of view, longer stents are more difficult to pass through microcatheters, because of increased friction between stent and catheter, and the trans-cell technique for coiling is more difficult to perform. A too-short stent may slip during deployment, resulting in incomplete coverage of the neck of the aneurysm, or may deploy into the aneurysm [13]. Although not as much of a challenge in laser cut stents as in braided stents, shortening of the stent during the procedure may exacerbate these difficulties [14]. Finally, if the stent diameter is too small, the stent may not cover the aneurysm neck, or may move or be difficult to deploy because of a lack of anchoring. Suboptimal stent placement in relation to the aneurysm may require the use of additional stents or retreatment.

Before the availability of stent planning applications using three-dimensional digital subtraction angiography (3D-DSA) images acquired by C-arm systems, stent selection would be performed by measuring the aneurysm and parent vessel dimensions on 2D fluoroscopic images. Stent selection was challenging, because curvatures of vessels and foreshortening on fluoroscopy images made it difficult to obtain accurate measurement of an artery segment’s length. In addition, prediction of the final location of a deployed stent of a specific selected length could be uncertain, as the stent may be distorted during deployment.

Recently developed stent planning software applications can now be used to assist the interventionalist in the optimal selection and placement of an intracranial stent. Based on a 3D-DSA image acquired before stenting, these applications simulate and display the final positioning of the deployed stent.

The accuracy of stent planning software has been extensively investigated for braided stents [15–19], but investigations are few on this subject for laser cut stents [20]. In this study, we aimed to evaluate the accuracy of a commercial software application for stent planning—in terms of difference in length between virtual and real stents—during SAC embolization of wide-necked aneurysms. We focused on laser cut stents and investigated whether the location of the aneurysm, the stent type, or the stent length had an effect on the difference in length between virtual and real stents.

## Materials and Methods

### Patient Population

The institutional review board of our hospital approved this study and waived informed consent because of its retrospective design. We reviewed data of 502 consecutive cases of unruptured aneurysm treated with coil embolization between July 2010 and March 2014. We restricted our study to patients with wide-necked aneurysms with a neck size ≥ 4 mm and a dome-to-neck ratio < 2 that were treated with the laser cut stents Enterprise (Cordis Neurovascular, Miami, FL, USA), a closed-cell, or Neuroform (Stryker, Kalamazoo, MI, USA) an open-cell stent, as these stent types can be visualized using available 3D-DSA. Of 84 eligible patients, nine patients were excluded because some or all stent markers were hidden by metal artifacts and thus not visible on 3D-DSA. The remaining 75 patients with 75 aneurysms were analyzed. Patient demographics and aneurysm characteristics are shown in Table 1.

**Table 1:** Characteristics of the sample

Patients (*n* = 75)	Frequency (%) or mean (SD)
*Sex*	
Male	18 (24%)
Female	57 (76%)
Mean age in years	58.8 (12.6)

Aneurysms and stents (*n* = 75)	
Mean aneurysm neck size in mm	7.2 (2.9)
Mean dome-to-neck ratio	1.1 (0.4)
*Stent location*	
Internal carotid	49 (65%)
Vertebral artery	18 (24%)
Basilar artery	7 (9%)
Middle cerebral artery	1 (1%)
*Aneurysm shape*	
Saccular	73 (97%)
Fusiform	2 (3%)
*Stent type*	
Enterprise	50 (67%)
Neuroform	25 (33%)
*Stent length*	
20 mm	20 (27%)
22 mm	11 (15%)
28 mm	36 (48%)
30 mm	4 (5%)
37 mm	4 (5%)
*Stent diameter*	
2.5 mm	1 (1%)
3 mm	2 (3%)
3.5 mm	1 (1%)
4 mm	8 (11%)
4.5 mm	63 (84%)

### SAC Embolization

SAC embolization was performed according to the standard of care at our institution, with the patient kept under general anesthesia. To prevent thrombosis, dual antiplatelet drugs were administered orally beginning 7 days before the SAC procedure. Systemic heparinization was administered as an initial 3000–5000 U bolus, followed by 1000 U/h under activated clotting time monitoring to maintain the clotting time at > 250 s throughout the procedure. An 8F balloon guiding catheter for the anterior circulation or a 7F guiding catheter for the posterior circulation was placed in the parent artery during embolization. Coil embolization was performed using a microcatheter with the jailing technique in all patients, and the stent was deployed across the neck of the aneurysm using virtual stent guidance (*syngo* 3D Aneurysm Guidance Neuro, Siemens Healthcare, Forchheim, Germany). Further coil packing was performed until a target volume embolization ratio of at least 20% was reached [21].

### Imaging Studies

All fluoroscopic and C-arm CT data were acquired on a biplane flat-panel detector angiography system (AXIOM Artis or Artis Q biplane, Siemens Healthcare GmbH, Forchheim, Germany). Reconstructions of the C-arm CT images and post-processing of the 3D-DSA images were performed on the system’s workstation (*syngo* X Workplace, Siemens Healthcare GmbH, Forchheim, Germany). Images were generated with a slice matrix of 256 × 256 (one case 512 × 512), an edge-enhanced reconstruction kernel, and an isotropic voxel size of 0.2–0.4 mm. 3D-DSA image data were acquired at the beginning and end of each procedure. Pre-procedural 3D-DSA images of the aneurysm and parent vessels were used for planning the procedure and selecting the stent. At the end of the procedure, fused images (*syngo* DualVolume, Siemens Healthcare GmbH, Forchheim, Germany) were used to confirm the coiling, the obliteration of the aneurysm, and the stent placement in the parent artery. These images consisted of non-contrast 3D images (mask images) fused with the 3D-DSA images of the vessels, resulting in 3D-DSA vessel images that included coils and stent markers.

### Stent Selection During the Procedure

All patients were treated by board-certified neurointerventionalists with 1–10 years experience. Stent length and placement decisions were made during the procedure by the treating interventionalist. Stents were selected by a standard procedure wherein the interventionalist selects a view of the vessel to be stented on 3D-DSA images with minimal foreshortening and measures the length and diameter of this vessel with electronic calipers. The results are cross-checked with the results of 2D-DSA images. The neck length of saccular wide-neck aneurysms or the segment length of fusiform aneurysms is measured. The stent length is selected to cover the aneurysm neck with an added length of a few millimeters in order to anchor the stent. Care is taken to avoid positioning the stent ends at bifurcating arteries.

For each patient, stents were also simulated with commercial stent planning software (*syngo* 3D Aneurysm Guidance Neuro, Siemens Healthcare GmbH, Forchheim, Germany). Based on 3D volume-rendered pre-procedural 3D-DSA images, the interventionalist clicks two points inside the aneurysm’s parent artery, one proximal and the other distal to the aneurysm, and a third point inside the aneurysm. The software application then automatically extracts and displays the parent artery’s centerline. The interventionalist manually corrects the centerline when needed. The stent is subsequently simulated along the parent artery based on the methods described previously [22]. The software application displays the virtual stent overlain on the volume-rendered 3D-DSA image. The interventionalist adjusts the virtual stent size to match the size of the real stent used for treatment and shifts the virtual stent along the parent vessel’s centerline as needed. The entire planning process takes approximately 2 min, with an additional 20 s of computer processing time.

Stents used in this study were Enterprise stents with a diameter of 4.5 mm and length of 22, 28, or 37 mm, and Neuroform stents with a diameter of 2.5, 3, 3.5, 4, or 4.5 mm and a length of 20 mm; or a diameter of 4 or 4.5 mm and a length of 30 mm.

### Measurements, Data Collection, and Analysis

For this retrospective study, all measurements were performed on a commercial workstation (*syngo* X Workplace, Siemens Healthcare GmbH, Forchheim, Germany) by one interventionalist (KN with 4 years of experience).

**Fig. 1:**
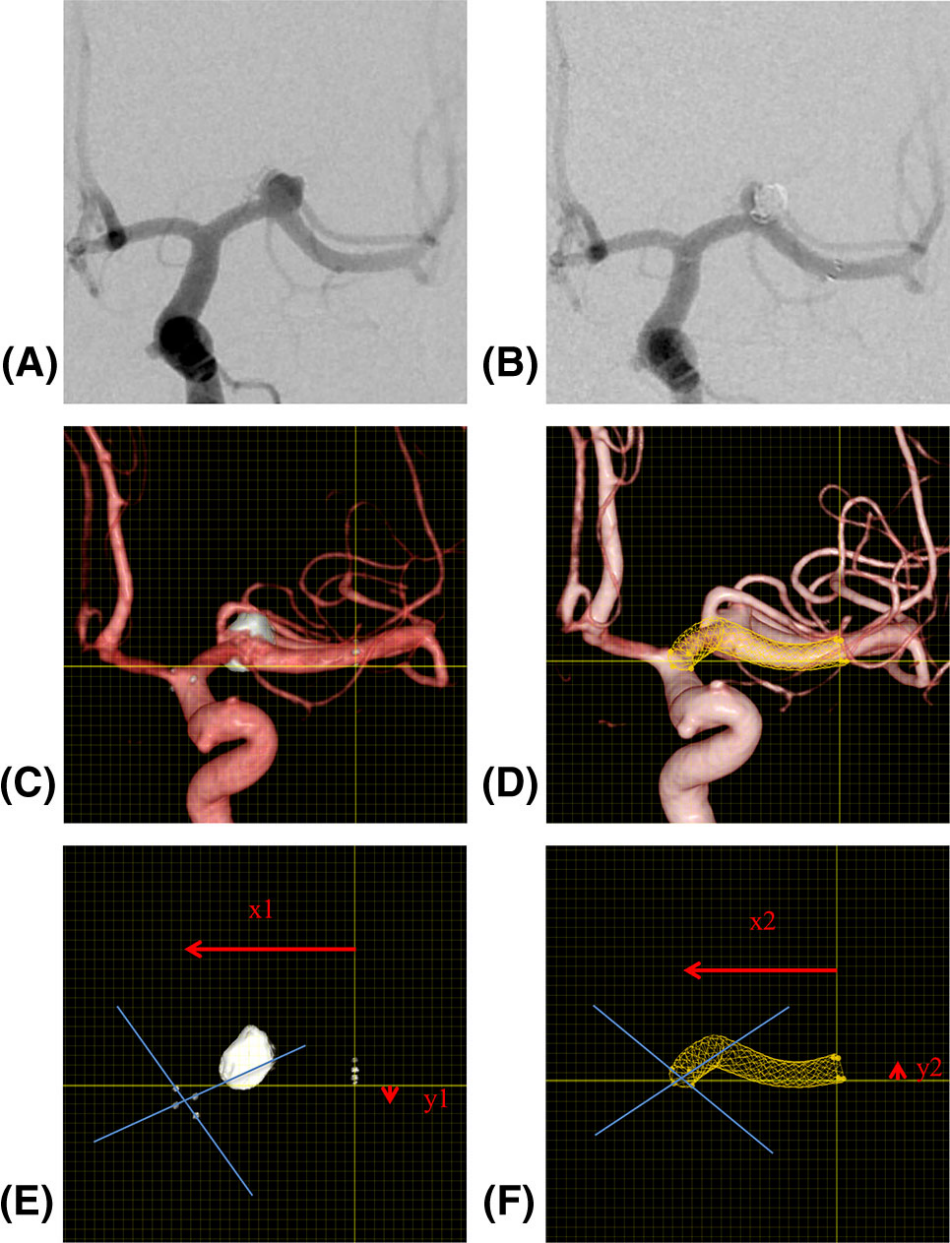
Example of an unruptured wide-necked aneurysm in the left middle cerebral artery (anterior circulation) treated with an open-cell stent 3 mm in diameter and 22 mm long, illustrating the discrepancies in measurement steps. **A** Angiography before treatment, 2D-DSA view used for stent selection. **B** Angiography of post-stent-assisted coiling of the same view as in **A**. **C** Post-procedural vascular 3D-DSA image including the real stent. Two lines were drawn through the real stent markers to define the proximal end point of the real stent. **D** Pre-procedural 3D-DSA vascular images. Two lines were drawn through the virtual stent markers to define the proximal end point of the virtual stent. **E** The coordinates (*X*_1_, *Y*_1_) of the real stent were recorded. **F** The coordinates (*X*_2_, *Y*_2_) of the virtual stent were recorded. The discrepancies d between end points of the virtual and real stents were calculated $d = \sqrt {\left( {X_{1} - X_{2} } \right)^{2} + \left( {Y_{1} - Y_{2} } \right)^{2} }$

The steps used to measure the difference in length between the stent end points are illustrated in Fig. 1. The pretreatment 2D-DSA images (Fig. 1A) with the least foreshortening of the vessel to be stented served as the reference for selecting the angles of all 3D images on which the measurements were performed. Figure 1B shows the 2D-DSA image acquired after treatment. First, the post-procedural vascular 3D-DSA images with the real stent were loaded into the viewing application of the workstation, and an angle was selected that corresponded to the view on 2D-DSA (Fig. 1B, C). The stent end points were defined by drawing two lines across the distal and proximal cross sections of the stent between two opposite markers with the drawing tools of the workstation (Fig. 1C). Then, the pre-procedural images were loaded, a stent was simulated, and the same steps were repeated to mark the virtual stent end points on the pre-procedural 3D-DSA vascular images (Fig. 1D). Finally, the post-procedural images were merged with the pre-procedural images and aligned for maximum congruence of the vessels while placing the distal ends of the virtual and real stents at the origin of a measuring grid displayed on the screen. The *x*- and *y*-coordinates of the end points of the virtual and real stent ends were recorded (Fig. 1E, F). The differences in length between the stents were calculated by applying Pythagoras’ theorem.

### Statistical Analysis

Statistical analyses were performed with commercial software (StataCorp. 2017. *Stata Statistical Software: Release 15*. College Station, TX: StataCorp LLC). Diagnostic plots were used to assess whether the distribution of the discrepancy measurements was normal. Since there was only one case of a stent in the middle cerebral artery, we added it to the stents in the basilar arteries for the statistical analysis, because of their similar dimensions. The Kruskal–Wallis test was used to test the null hypothesis that there is no effect of stent location, stent type, or stent length on the discrepancy between virtual and real stents.

## Results

**Fig. 2:**
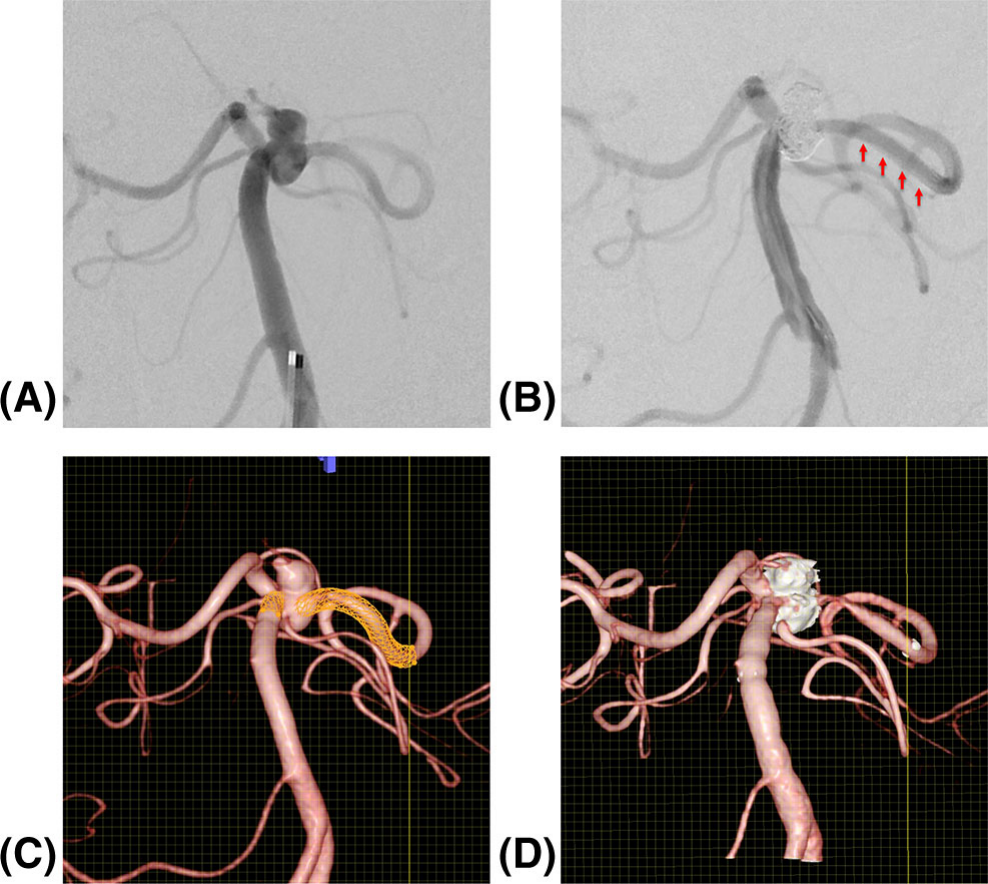
An unruptured wide-necked basilar tip aneurysm treated with a closed-cell stent 4.5 mm diameter and 28 mm long in the posterior circulation. This case had the largest discrepancy, 6.18 mm, of all virtual stent/real stent pairs. **A** Angiography before treatment. **B** Angiography of post-stent-assisted coiling with the same view as in **A**. Arrow shows that the shape of the parent vessel has changed. **C** Pre-procedural 3D-DSA vascular images with virtual stent. **D** Post-procedural vascular 3D-DSA image including the real stent

The simulation software application successfully generated virtual stents for all patients. The stents in all 75 patients were successfully deployed, and all stents covered the necks of the aneurysms. The discrepancy between distal stent ends was small, as expected, because the virtual and real distal stent ends were manually aligned through the procedure described above. However, for stents that were not perfectly deployed, stent markers did not line up on a perpendicular plane relative to the vessel wall. In these cases, we choose to adjust the end of the stents to be as congruent as possible while compromising the alignment of the cross-point of the stent markers and the center point of the virtual stent end. The discrepancy of the distal stent end points thus ranged from 0 to 2.69 mm, with a median of 0.50 mm and interquartile range (IQR) of 0.50–1.00 mm. The difference in length measured between the proximal stent end points ranged from 0 to 6.52 mm; the median difference in length was 1.58 mm with IQR of 1.00 mm, or, relative to the length of the stents, a median of 6%, IQR of 5–9%, and range of 0–22%. The real stent appeared shorter than the virtual stent for 12 stents (median 1.58 mm, IQR 1.41–2.03 mm), longer for 55 stents (mean 1.80 mm, IQR 1.41–2.50 mm), and very similar for 8 stents (median 0.50 mm, IQR 0.25–1.00 mm), where the discrepancy was caused by the stent markers not perfectly deployed, but the stents being congruent. All the 12 shortened stents were located in the internal carotid artery. Differences in length of > 4.0 mm were found for four Enterprise stents, namely 4.5 mm for a 22 mm stent in the paraclinoid internal carotid artery, 4.61 mm for a 28 mm stent in the paraclinoid internal carotid artery, 4.61 mm for a 37 mm stent in the posterior communicating internal carotid artery, and 6.18 mm for a 28 mm stent in the basilar artery. In these cases, the real stent appeared longer than the virtual stent. Inspection of the images revealed that the shape of the parent vessel changed before and after stenting (Fig. 2). As a result, the virtual and real stents were not precisely congruent, leading to the discrepancies in their lengths.

**Fig. 3:**
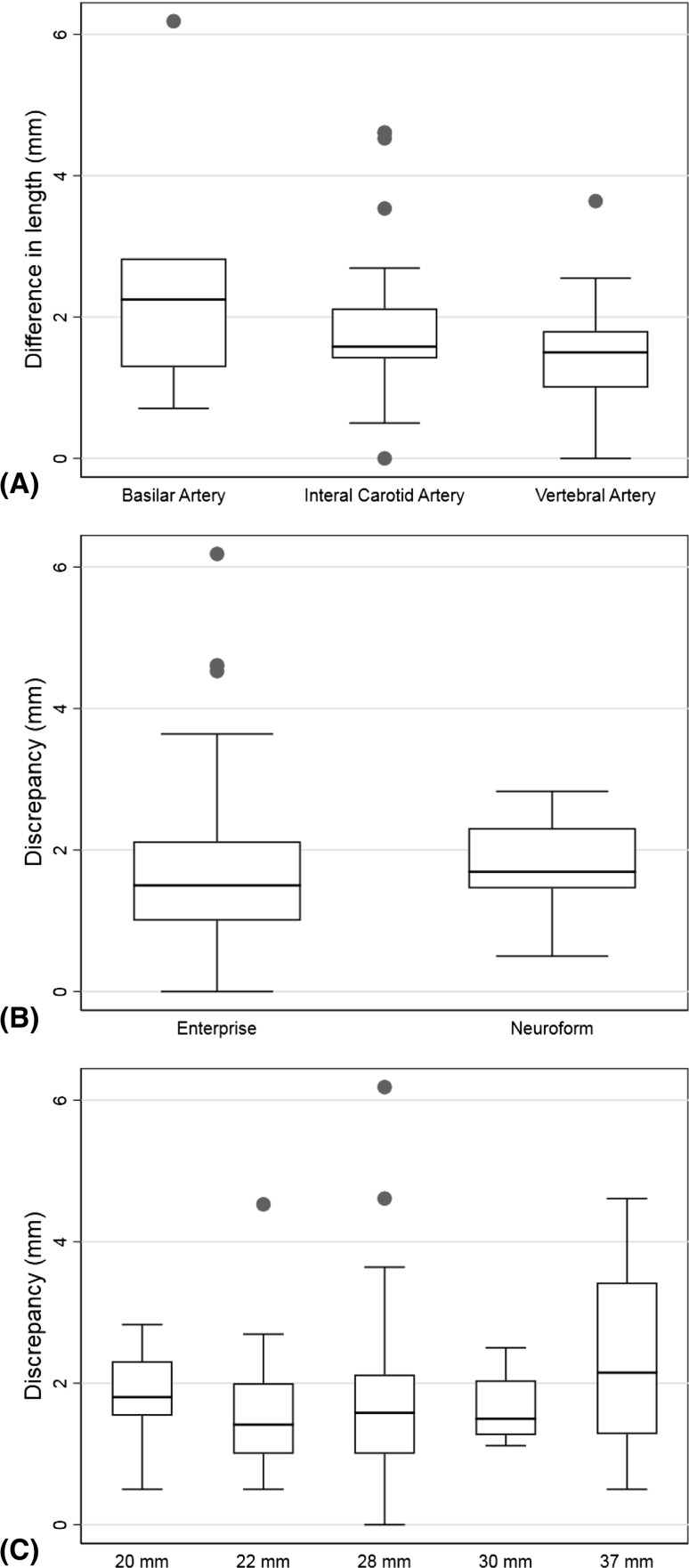
Box plots of the differences in length by **A** location of the aneurysm, **B** type of stent, **C** stent length (**C**)

The Kruskal–Wallis test results showed no evidence for an effect on difference in length of location of the aneurysm (*p *= 0.23) or for type of stent (*p *= 0.33) or for stent length (*p *= 0.53). Figure 3 shows box plots of the differences in length by stent location, type of stent, and stent length.

## Discussion

The stent planning software application generated 3D images of virtual stents overlain on 3D-DSA vessel images for all cases. The real stent deployments were all successful and had appropriate sizes and positions in terms of deployment inside the vessel and coverage of the aneurysm neck. This applied to the laser cut stents Enterprise and Neuroform of a wide range of lengths and diameters for aneurysms with parent vessels of various shapes located in the anterior or posterior circulation, suggesting that the software application could simulate appropriate virtual stents even for aneurysms with tortuous parent arteries.

Small differences in length could be expected to be caused by deformation of the real stent during deployment which differed from that of the virtual stent [14], particularly as the software application did not model the mechanical behavior of the stent in terms of stent shortening. Real stents may also slightly bulge into the aneurysm in the case of large aneurysms, which may contribute to shortening of the real stent length.

We found that for tortuous parent arteries or large aneurysms the software application generated centerlines that needed more manual correction than those in straighter vessels and for smaller aneurysms. Some degree of error may thus have been introduced by the centerline segmentation.

In our study, the end points of the stent were determined using the intersection of lines drawn through the stent markers. For the virtual stent, this resulted in a precise center point, because the virtual stents were uniformly deployed in all directions radial to the vessel. However, the real stent flare does not always open uniformly radial to the vessel and the markers of the stent therefore do not necessarily expand to equal extents. This may have introduced a shift in the real stent end point and an error in the discrepancy measurements.

We found the largest discrepancies between virtual and real stents in cases in which the real stent seemed to have caused deformation of the vessel. It has been reported that for closed-cell stents, the radial expansion force and closed-cell design may not allow full expansion, especially at high curvatures [23, 24], and that significant straightening of arteries could occur due to stent deployment [14, 25, 26]. Vessel straightening and shortening of the virtual stent are not taken into account by the stent planning software application, which could explain the straightening vessels seen in our study for the cases with the largest discrepancies, especially in the posterior fossa, where the diameters of the parent artery are small. For all large discrepancies, the real stent appeared longer than the virtual stent due to deformation of the parent vessel, which indicates that the virtual stents generated by the software application may be safely used.

Our study has limitations. Comparisons of the virtual and real stents were carried out in 2D and not in 3D along its full length; accordingly, the difference in length only represents a 2D projection of the difference in length or position of the stent and foreshortening of vessels may have introduced errors. It should also be noted that 2D images are still routinely used in surgery. Only the stent end markers were visible on 3D-DSA images, and discrepancies between virtual and real stents could not be evaluated at other locations or along the whole stent. Some stents were not perfectly deployed and the cross-point of the stent markers did not match the center point of the stented vessel. We did not correct for this discrepancy in the length calculation, because it would have introduced a shift of the whole stent and an error in the real stent measurement. This introduced small discrepancies between virtual and real stent end at the distal stent end points. The presence of coils introduced metallic artifacts, which partly impaired stent visualization. We investigated only Enterprise and Neuroform stents. Braided stents such as LVIS (Microvention Terumo, California, USA) and Pipeline (Medtronic, Minnesota, USA) shrink by design during deployment and could not be simulated with this software application.

## Conclusion

The results of this study suggest that stent planning software is a useful tool for stent planning in SAC embolization of intracranial aneurysms. The software application allows 3D simulations of stents overlain on 3D-DSA images of vessels and led to acceptable results in terms of discrepancy between virtual and real stent location for laser cut stents.

## Abbreviations

2D: Two dimensional
3D: Three dimensional
DSA: Digital subtraction angiography
SAC: Stent-assisted coiling
